# A Novel Deleterious MYO15A Gene Mutation Causes Nonsyndromic Hearing Loss

**DOI:** 10.22038/IJORL.2023.69889.3372

**Published:** 2024-01

**Authors:** Mostafa Neissi, Adnan Issa Al-Badran, Javad Mohammadi-Asl

**Affiliations:** 1 *Department of Genetics, Khuzestan Science and Research Branch, Islamic Azad University, Ahvaz, Iran. Department of Genetics, Ahvaz Branch, Islamic Azad University, Ahvaz, Iran.*; 2 *Department of Biology, College of Science, University of Basrah, Basrah, Iraq.*; 3 *Department of Medical Genetics, School of Medicine, Ahvaz Jundishapur University of Medical Sciences, Ahvaz, Iran.*

**Keywords:** Hearing loss, MYO15A gene, Mutation

## Abstract

**Introduction:**

Hearing loss (HL) is the most frequent sensory neurodeficiency, affecting a broad spectrum of individuals globally. Within this context, the role of genetic factors takes center stage, particularly in cases of hereditary HL.

**Case Report:**

Here, we present a nonsyndromic HL (NSHL) case report. The patient is a 21-year-old man with progressive HL. The whole-exome sequencing (WES) demonstrated a novel homozygous missense mutation, c.9908A>C; p.Lys3303Thr, in the proband's exon 61 of the MYO15A gene. Further analysis has revealed that the detected mutation is present in a heterozygous state in the parents.

**Conclusion:**

WES analysis in this study revealed a novel mutation in the MYO15A gene. Our data indicates that the MYO15A-p.Lys3303Thr mutation is the likely pathogenic variant associated with NSHL. Additionally, this finding enhances genetic counseling for individuals with NSHL patients, highlighting the value of the WES method in detecting rare genetic variants.

## Introduction

Hearing loss (HL) is considered the most common sensorineural disorder, and according to the World Health Organization (WHO), it affects over 5% of the world's population (1). Genetic factors are responsible for almost half of all recognized impairments. Hearing impairment has two types: syndromic and nonsyndromic HL (NSHL) classification, the latter being highly heterogeneous. In the syndromic type, deafness is conductive HL and is accompanied by further dysfunctions. In contrast, in NSHL, the clinical manifestation is just deafness, and the disorder is sensorineural HL type (2–4). NSHL is an inherited condition primarily involving just the auditory sensation; it accounts for 70% of all inherited HL cases. NSHL is inherited as autosomal recessive (ARNSHL) and autosomal dominant (ADNSHL) inheritance forms. 80% of gene deficits of hearing impairments are expected to be ARNSHL, and the remaining 20% ADNSHL (1,2). ARNSHL is primarily caused by genes encoding myosin-XVa (MYO15A), solute carrier family 26 (anion exchanger) member 4 (SLC26A4), gap junction protein beta 2 (GJB2), otoferlin (OTOF), cadherin-related 23 (CDH23), and transmembrane channel- like 1 (TMC1), all of which contain over 20 deferent mutations that have been identified in consanguineous marriage (5). It is estimated that more than 150 genes are involved in hearing impairment, accounting for the heterogeneity of HL. HL highly influences the life quality of patients. It was demonstrated that HL is accompanied by dementia, reduction in functional abilities, cardiovascular disease and higher mortality (6–8). HL can be detected in the early stages of growth or may identified in the elderly stages. The most frequent pattern of hereditary HL (HHL) is autosomal recessive (85%), followed by autosomal dominant (12-15%) and X-link (1-3%), respectively (9). In regions where consanguinity marriage has a high rate, identifying rare pathogenic mutations is not far from expected. In Iran, the rate of consanguinity marriage was reported to be approximately 40%, and it is estimated that it will reach 60% in the future (2), which indicates the importance of reliable gene patterns. In this study, we report a new mutation in the MYO15A gene, which is involved in the pathogenesis of NSHL.

## Case Report

**Fig 1:**
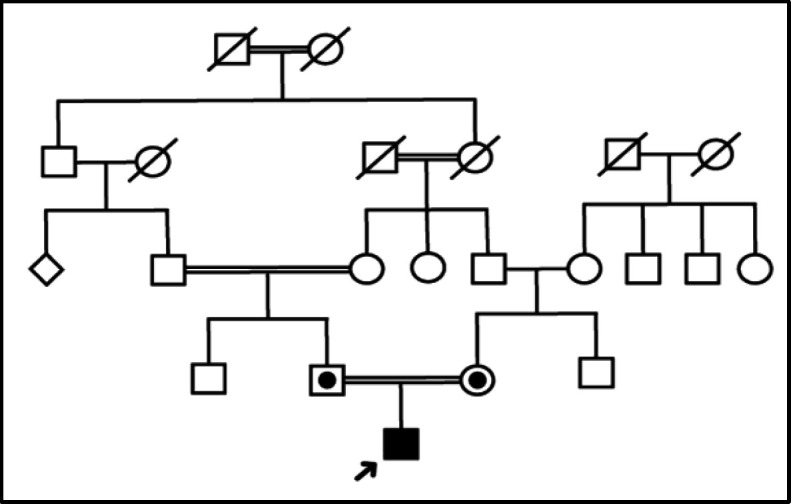
Pedigree of the studied family with an autosomal recessive pattern of inheritance.

We genetically analyzed an Iranian family with a consanguineous marriage with a 21-year-old boy with HHL. The family pedigree is depicted in Figure 1. The blood specimens were obtained from all family members. All available medical history was collected. The otolaryngologist evaluated all family members. 

Circle symbols represent females, squares denote males and rhombus indicate gender unknown. The diagonal line on the squares and circles represents a deceased male and female. Filled black symbols represent the patient, and symbols with dots represent mutation carriers without clinical manifestations. The arrow indicates the proband of this family.

It was determined that the subject had sensorineural HL (SNHL) based on pure tone audiometry (PTA). There were no clinical manifestations in favor of the syndromic phenotype. DNA extraction was conducted using the salting out method. We solely performed whole-exome sequencing (WES) for the proband. DNA sequencing was carried out by SureSelect Human All Exon Kit V6 (Agilent Technologies Inc., USA) and Illumina HiSeq 4000 machine (San Diego, USA) in accordance with the manufacturer's instructions. Genetic sequence analysis detected a novel, homozygous substitution at c.9908A>C (NM_016239.4) in exon 61 of the MYO15A gene. Bioinformatic tools, including SIFT, PolyPhen-2, and MutationTaster, predicted that this mutation is probably pathogenic (Table 1).

**Table 1:** Results of in silico prediction tools for the functional effect of the novel missense mutation.

Gene/variant	Polyphen-2 HDIV score	SIFT score	MutationTaster
ENST00000647165.2, K3303T	1.000 (probably damaging)	0.007 (damaging)	Disease-causing

**Fig 2:**
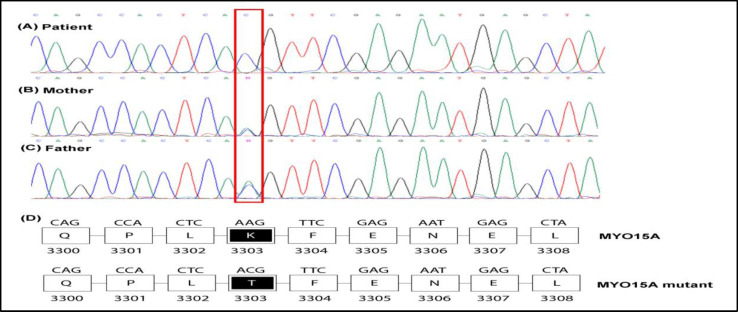
Sanger sequencing chromatograms of chromosome 17 at position 18166481 for the patient (A) and his parents (B,C) are shown.

The detected mutation in the proband was verified using Sanger direct sequencing (ABI 3130 Genetic Analyzer, California, USA). The sequences of used primers were as follows (forward primer: AAGCTGTGTCCCAGAAC AGG and the reverse primer ACAGGGCCT GAATCATGA AC). Figure 2 shows that the patient and his parents had the MYO15A mutation in the homozygous and heterozygous states, respectively. This missense mutation substitutes Lysine with Threonine (AAG>ACG) at the 3303-position of the MYO15A protein (Figure 2D). These observations suggest that NM_016239.4 (MYO15A): c.9908A>C; p. Lys3303Thr mutation could be the cause of the progress of NSHL. The detected mutation information is shown in Table 2 based on the Human Gene Mutation Database (10).

The nucleotide changes A to C at chr17:18166481 which results in MYO15A p.Lys3303Thr are highlighted by boxes. The DNA sequence chromatogram of the patient shows homozygous C/C at chr17:18166481 while the chromatogram of his parents shows heterozygous A/C. (D) Zoomed-in view of the region containing the K3303T variant, including the amino acid sequences of protein-coding isoform and the mutated sequence caused by the variant.

**Table 2:** Reported mutations in MYO15A gene.

Pathogenic variant	Protein effect	Exon	Type of mutation
c.742C>G	p.Arg248Gly	2	Missense
c.867C>G	p.Tyr289Ter	2	Nonsense
c.1047C>G	p.Tyr349Ter	2	Nonsense
c.1210G>T	p.Glu404Ter	2	Nonsense
c.1223C>T	p.Ala408Val	2	Missense
c.1387A>C	p.Met463Val	2	Missense
c.3026C>A	p.Pro1009His	2	Missense
c.3313G>T	p.Glu1105Ter	2	Nonsense
c.3385C>T	p.Arg1129Ter	2	Nonsense
c.4351G>A	(p.Asp1451Asn)	12	Missense
c.4652C>A	p.Ala1551Asp	14	Missense
c.4898T>C	p.Ile1633Thr	17	Missense
c.5189T>C	p.Leu1730Pro	19	Missense
c.5336T>C	p.Leu1779Pro	20	Missense
c.5417T>C	p.Leu1806Pro	22	Missense
c.9620G>A	p.Arg3207His	59	Missense
c.10263C>G	p.Ile3421Met	64	Missense
c.10474C>T	p.Gln3492Ter	65	Missense

## Discussion

In the present study, we analyzed an affected person with HL using the WES method. Our results indicated a novel variant of the MYO15A gene in patients with ARNSH. This gene deficit was a new homozygous mutation, c.9908A>C in exon 61, that cause converting the Lysine at position 3303 to Threonine (p.K3303T) in the ferm domain and tail region of the Myosin protein. This alteration is accompanied by the production of a polar uncharged amino acid instead of a polar charged amino acid. Based on in silico predictions, this change will negatively affect myosin-XVa protein function. Up to now, the Human Gene Mutation Database has presented 241 missense/nonsense mutations in the MYO15A gene (10); the data of our study has added a new missense MYO15A gene mutation to the current knowledge.

Myosin-XVa, the coding protein of MYO15A, plays a critical role in forming stereocilia in the hair cells of the cochlea (11). As an actin-activated ATPase that uses ATP hydrolysis to move on actin filaments, myosin XVa is completely localized at the tips of stereocilia in the organ of Corti. It is believed that mechano-electric transduction occurs at the tip of a stereocilium and the site of stereocilia growth (12). It is crucial for the formation and proper function of the mechanotransduction machinery to have myosin-XVa. As myosin tails bind with membranous compartments, they move relative to actin filaments (13). Also, our work suggests that the autosomal recessive form of NSHL could be a consequence of MYO15A gene mutations and c.9908A>C MYO15A mutation encodes a dysfunction of MYO15A protein that causes function or stability, and finally deafness. Wang L et al. in their publication, showed causative MYO15A mutations in a Chinese family with NSHL (14). Furthermore, previous studies have evaluated pathogenic genomic defect families with NSHL, introduced forty-three mutations in the MYO15A gene, and concluded that modifications accounted for ARNSHL (14–16). Subsequently, Zhang F. et al. reported three MYO15A pathogenic mutations (c.3971C>A; p.A1324D, c.4011insA; p.Q1337Qfs∗22, and c.9690+1G>A) in one Chinese family with ARNSHL (17). In line with these findings, Asgharzade S. et al. evaluated the involved mutations of NSHL in the Arab population in Southwest Iran using WES and presented a novel homozygous c.1047C>A (p.Y349∗) mutation in one of the twenty-five families that produce a premature stop codon (18). European epidemiological data revealed that RNSHL, DFNA22, DFNA8/12, and ADNSHL, DFNB1 are more prevalent (19). Additionally, they indicated that maternally inherited X-linked HL NSHL have the lowest frequency of hereditary patterns (19). Despite recognizing various gene mutations, they account for approximately 50% of HL in Europe (20). These findings indicated a challenge in HL gene mutation diagnosis. In a meta-analysis conducted by Farjami M et al. investigated the prevalence of MYO15A gene mutations around the world, it was found that the MYO15A gene is one of the most causative gene deficits in NSHL and it is highly dependent on the ethnic background (21). In this line, it was demonstrated that in the United Arab Emirates (UAE), missense mutations are more frequent in NSHL pathogenesis, in which the most involved genes were GJB2 and CDC14A (22). It agrees with Adadey SM et al.'s findings that connexin gene variants are prevalent in NSHL, although they have indicated biallelic GJB2 likely pathogenic were more common (23). This is the first report of c.9908A>C mutation in the MYO15A gene in NSHL patients. Since only this mutation was detected by WES and confirmed by Sanger sequencing, it can be concluded that it can cause NSHL. It is postulated that variant chr17:18166481:A>C (in exon 61 of MYO15A gene) in the ferm domain and tail region of the Myosin protein can create a substitution in the amino acid sequence and produce a new codon at position 3303. This alteration would be expected to affect the MYO15A protein's function. This mutation in the MYO15A gene is likely pathogenic in our patient affected with NSHL.

## Conclusion

 The present study reported a novel missense mutation (c.9908A>C; p.Lys3303Thr) in the MYO15A gene. This information holds considerable significance within the realm of genetic counseling, particularly for individuals diagnosed with NSHL. Furthermore, we demonstrate the effectiveness of the WES method as a valuable tool for identifying rare causative genetic variants in patients with NSHL.

## References

[1] Deafness and hearing loss [Internet].

[2] Mohammadi-Asl J, Saki N, Karimi M, Ghanbari Mardasi F (2021). Identification of a Novel Frameshift Mutation in the TECTA Gene in an Iranian Family With Autosomal Nonsyndromic Hearing Loss. Acta Med Iran..

[3] Mohammadi-Asl J, Saki N, Dehdashtiyan M, Neissi M, Ghanbari Mardasi F (2021). Identification of a Novel WFS1 Mutation Using the Whole Exome Sequencing in an Iranian Pedigree with Autosomal Dominant Hearing Loss. Iran J Otorhinolaryngol..

[4] Neissi M, Abdulzahra HKh, Sheikh-Hosseini M, Mabudi H, Mohammadi-Asl J, Al-Badran RA (2022). Homozygous LOXHD1 Nonsense Mutation (c 1787G>A; p W596X) is Associated with Hearing Loss in an Iranian Family: A Case Report. Int J Biomed..

[5] Hilgert N, Smith RJH, Van Camp G (2009). Forty-six genes causing nonsyndromic hearing impairment: which ones should be analyzed in DNA diagnostics?. Mutat Res..

[6] Dixon PR, Feeny D, Tomlinson G, Cushing S, Chen JM, Krahn MD (2020). Health-Related Quality of Life Changes Associated with Hearing Loss. JAMA Otolaryngol Head Neck Surg..

[7] Griffiths TD, Lad M, Kumar S, Holmes E, McMurray B, Maguire EA (2020). How Can Hearing Loss Cause Dementia?. Neuron..

[8] Lee W, Chang Y, Shin H, Ryu S (2020). Hearing Loss and Risk of Overall, Injury-Related, and Cardiovascular Mortality: The Kangbuk Samsung Health Study. J Clin Med..

[9] Aldè M, Cantarella G, Zanetti D, Pignataro L, La Mantia I, Maiolino L (2023). Autosomal Dominant Non-Syndromic Hearing Loss (DFNA): A Comprehensive Narrative Review. Biomedicines..

[10] HGMD® home page [Internet].

[11] Anderson DW, Probst FJ, Belyantseva IA, Fridell RA, Beyer L, Martin DM (2000). The motor and tail regions of myosin XV are critical for normal structure and function of auditory and vestibular hair cells. Hum Mol Genet..

[12] Belyantseva IA, Boger ET, Friedman TB (2003). Myosin XVa localizes to the tips of inner ear sensory cell stereocilia and is essential for staircase formation of the hair bundle. Proc Natl Acad Sci U S A..

[13] Wang A, Liang Y, Fridell RA, Probst FJ, Wilcox ER, Touchman JW (1998). Association of unconventional myosin MYO15 mutations with human nonsyndromic deafness DFNB3. Science..

[14] Wang L, Zhang Y, Xue Q, Huang P, Liu X (2022). Identification of novel compound heterozygous mutations of the MYO15A gene with autosomal recessive non-syndromic hearing loss. J Clin Lab Anal..

[15] Cengiz FB, Duman D, Sirmaci A, Tokgöz-Yilmaz S, Erbek S, Oztürkmen-Akay H (2010). Recurrent and private MYO15A mutations are associated with deafness in the Turkish population. Genet Test Mol Biomarkers..

[16] Bashir R, Fatima A, Naz S (2012). Prioritized sequencing of the second exon of MYO15A reveals a new mutation segregating in a Pakistani family with moderate to severe hearing loss. Eur J Med Genet..

[17] Zhang F, Xu L, Xiao Y, Li J, Bai X, Wang H (2018). Three MYO15A Mutations Identified in One Chinese Family with Autosomal Recessive Nonsyndromic Hearing Loss. Neural Plast..

[18] Asgharzade S, Chaleshtori MH, Tabatabaifar MA, Reisi S, Modaressi MH (2016). Mutation in second exon of MYO15A gene cause of nonsyndromic hearing loss and its association in the Arab population in Iran. Genetika.

[19] Del Castillo I, Morín M, Domínguez-Ruiz M, Moreno-Pelayo MA (2022). Genetic etiology of non-syndromic hearing loss in Europe. Hum Genet..

[20] Vona B, Doll J, Hofrichter MAH, Haaf T (2020). Nonsyndromic hearing loss: Clinical and diagnostic challenges. Med Genet..

[21] Farjami M, Assadi R, Afzal Javan F, Alimardani M, Eslami S, Mansoori Derakhshan S (2020). The worldwide frequency of MYO15A gene mutations in patients with non-syndromic hearing loss: A meta-analysis. Iran J Basic Med Sci..

[22] Elsayed O, Al-Shamsi A (2022). Mutation spectrum of non-syndromic hearing loss in the UAE, a retrospective cohort study and literature review. Mol Genet Genomic Med..

[23] Adadey SM, Wonkam-Tingang E, Twumasi Aboagye E, Nayo-Gyan DW, Boatemaa Ansong M, Quaye O (2020). Connexin Genes Variants Associated with Non-Syndromic Hearing Impairment: A Systematic Review of the Global Burden. Life (Basel)..

